# A hybrid Bayesian network-based deep learning approach combining climatic and reliability factors to forecast electric vehicle charging capacity

**DOI:** 10.1016/j.heliyon.2025.e42483

**Published:** 2025-02-14

**Authors:** David Chunhu Li

**Affiliations:** Information Technology and Management Program, Ming Chuan University, Taoyuan City, 33321, Taiwan, ROC

**Keywords:** Electric vehicle infrastructure, Charging station reliability, Predictive modeling, Deep learning, Environmental impact, Smart grid optimization

## Abstract

The increasing adoption of electric vehicles (EVs) necessitates advanced predictive models to accurately forecast charging demand and ensure reliable infrastructure planning. This study introduces a novel analytical framework that integrates queuing network and Bayesian network models to enhance the prediction accuracy and reliability of EV charging demand. The objective is to develop a comprehensive system that accounts for various influencing factors, such as meteorological conditions and charging pile failure rates, to optimize EV infrastructure. The methodology involves creating a hybrid Bayesian Network-based deep learning (HBNDL) system architecture. This architecture uses extensive transaction data and climate analysis to build a detailed model of EV charging pile reliability. Additionally, two algorithms are designed to assess the usage and reliability of charging stations. The framework's effectiveness is tested through a series of experiments evaluating its performance in short-, medium-, and long-term prediction scenarios. The results demonstrate that the HBNDL framework significantly improves prediction accuracy and infrastructure reliability. The integration of queuing theory and Bayesian network models with deep learning techniques results in a robust system adaptable to various conditions. Experimental validation shows that the proposed framework outperforms existing models in forecasting EV charging demand, particularly under varying environmental influences.

## Highlights

• Developed a hybrid Bayesian Network-based deep learning framework for EV demand prediction.
• Found a negative correlation between rainfall and EV charging demand for infrastructure optimization.
• Introduced algorithms to assess EV station usage and reliability, improving infrastructure.

## Graphical abstract

**Figure:**
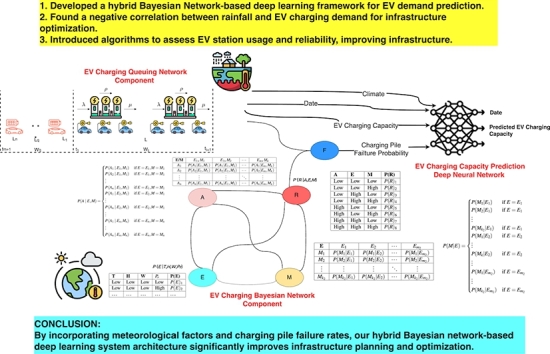

## 1 Introduction

The adoption of electric vehicles (EVs) has experienced significant growth in recent years, driven by increasing awareness of environmental concerns and the pursuit of sustainable transportation solutions. As the world progresses toward a low-carbon future, EVs have emerged as a promising substitute for conventional internal combustion engine vehicles, offering reduced greenhouse gas emissions and improved energy efficiency [1]. However, the widespread integration of EVs into global transportation infrastructures presents several challenges that must be addressed to ensure a seamless and efficient transition [2].

With the growing adoption of EVs, developing accurate and reliable models for predicting EV charging demand has become a key research area. Recent studies have focused on integrating machine learning algorithms, big data analytics, and advanced forecasting techniques to improve prediction accuracy [3], [4], [5]. Incorporating factors such as environmental conditions, user charging behaviors, and real-time energy availability is critical for building robust systems that forecast charging demand effectively [6], [7]. These advancements have contributed to the improvement of predictive models, which are increasingly relied upon to optimize EV infrastructure deployment and manage charging load distribution.

One of the most critical challenges associated with the widespread adoption of EVs is the demand for an extensive and reliable charging infrastructure [8], [9]. To meet the charging needs of a growing number of EVs, understanding and accurately predicting the charging demand are essential. Failure to anticipate and accommodate this demand could result in infrastructure overload, increased charging time, and potential inconvenience to EV owners [10], [11]. Consequently, deployment optimization for charging infrastructures and strategy development to manage charging demands are of paramount importance. The analysis and prediction of EV charging demand are complex tasks that require the examination of numerous interrelated factors [12], [13].

Despite significant advancements in predicting EV charging demand, several challenges persist in developing models that can accurately forecast charging needs under diverse conditions. First, the variability in user charging behavior and preferences introduces uncertainty into prediction models [14]. Second, environmental factors such as extreme temperatures and precipitation can significantly affect battery performance and charging station availability, complicating accurate demand forecasts [15]. Third, the aging of charging infrastructure components and the likelihood of charging pile failures further increase uncertainty in predicting charging needs [16]. These challenges underscore the necessity for more integrated models that account for both operational and environmental factors to improve prediction accuracy.

To address this challenge, this study proposes a novel hybrid Bayesian network model that integrates queuing theory with Bayesian networks. The model considers factors such as the aging of charging infrastructure components, environmental conditions, and maintenance activities to calculate the failure rate of EV charging piles accurately. By leveraging the designed hybrid Bayesian network model, we aim to improve the accuracy and rationality of predicting electric vehicle charging demand. The calculated failure rate of EV charging piles serves as a crucial input parameter for training a deep learning prediction model. This integration of advanced modeling techniques not only enhances the accuracy of predicting charging demand but also ensures the reliability and efficiency of EV charging infrastructure.

Meteorological conditions, including temperature, humidity, and precipitation, can significantly influence EV charging patterns. For instance, extreme temperatures can impact battery performance and alter drivers' behaviors, which can lead to fluctuations in charging demand [17], [18], [19]. In addition, weather conditions can affect the availability and efficiency of renewable energy sources, which are increasingly being integrated into power grids to support sustainable charging. In charging demand analyses, the consideration of meteorological factors can enhance the accuracy of predictions and enable the proactive management of charging infrastructures [20], [21], [22].

The reliability and uptime of charging infrastructures are crucial for providing a seamless charging experience to EV users. Charging pile failures, such as equipment malfunction or power supply interruptions, can disrupt the charging process and lead to dissatisfied customers [23]. Understanding the factors that contribute to charging pile failures can enable proactive maintenance, reduce downtime, and improve the overall reliability of charging networks. Meanwhile, the integration of charging pile failure analysis with charging demand prediction can provide valuable insights for efficient infrastructure planning and maintenance scheduling.

The primary objective of this research is to analyze and predict EV charging demand by incorporating meteorological and charging pile failure factors into the analysis. By considering the additional variables, we aim to enhance the accuracy and reliability of charging demand predictions for improved infrastructure planning and optimization. The main research contributions of this study are as follows:

(1) We present a comprehensive system model that integrates queuing and Bayesian network models to estimate the reliability of EV charging piles, taking into account meteorological factors and charging pile failures.
(2) We design two algorithms: one to evaluate the usage of electric vehicle charging stations using a queuing network model, and the other to integrate the Bayesian network and queuing network models to calculate the reliability of charging piles.
(3) We leverage transaction data from EV charging stations in Palo Alto, California, the United States of America, spanning the last decade, as a case study. Employing big data analytic techniques, we investigate the influence of climatic factors on demand for EV charging capacity.
(4) We propose a hybrid Bayesian network-based deep learning (HBNDL) system architecture that integrates a queuing network, a Bayesian network and deep neural networks to develop a model-training framework. We train a set of deep learning models, categorized into five groups, using the HBNDL framework to predict EV charging demand in short-, medium-, and long-term scenarios. The extensive experimentation validates the substantial adaptability and robustness of the HBNDL and reinforces its effectiveness.

The remainder of this paper is structured as follows: Section 2 reviews prior research on EV charging capacity prediction, Section 3 introduces the proposed system model and outlines the algorithms for evaluating the reliability of EV charging stations, Section 3 also delves into the use of a big data analysis model to examine the influence of climate factors on EV charging capacity demand, Section 4 details the experiment analysis and discusses the corresponding results, and finally, Section 5 concludes the study and outlines potential avenues for future research.

## 2 Related works

The charging capacity of EVs pertains to the amount of electric power needed to recharge the battery from a depleted state to the desired level. It can directly influence the efficiency and reliability of the charging process and overall accessibility and utilization of charging infrastructures. The prediction of EV charging capacity emerged progressively as a significant research area, given its profound implications for the seamless and optimized operation of charging networks, enhancement of user satisfaction, and effective energy management [24], [25], [26].

Jiang et al. [27] introduced a novel modeling framework for predicting public charging demand profiles for EVs based on people's travel trajectories. The methodology explicitly models the decision-making process for vehicle charging and estimates the charging needs of individual EV users. By leveraging activity-based and agent-based transportation simulation models, as well as employing a scenario-based approach, their study captured variations in both the demand and supply sides of transportation systems. The paper [28] presented a novel approach using Long Short-Term Memory (LSTM) neural networks for short-term prediction of EV charging demand, focusing on the next few hours. Leveraging a comprehensive trajectory dataset of over 76,000 private EVs in Beijing, this study explored LSTM's performance against traditional models like Autoregressive Integrated Moving Average model (ARIMA) and Multilayer Perceptron model (MLP). Results indicate LSTM's superiority, with significantly lower Mean Absolute Percentage Error (MAPE) values. The study in [29] pioneered the application of the Transformer model in forecasting EV charging demand, addressing both short-term and long-term periods. Leveraging real-world EV charging records from 25 public stations in Boulder, Colorado, this research compared Transformer's performance with traditional and machine-learning models such as ARIMA, seasonal ARIMA (SARIMA), LSTM, and Recurrent Neural Network (RNN). Results indicated that Transformer consistently outperforms these models, particularly in long-term predictions.

A model-based multichannel convolutional neural network and temporal convolutional network (MCCNN-TCN) framework was proposed in [30] for predicting the charging load of EVs within 7 days, which consisted of two parts: the MCCNN, which is a multichannel convolutional neural network for extracting the characteristics of EV charging load fluctuations over various time lengths, and TCN, which is a temporal convolutional neural network for establishing a time series between the fluctuation characteristics of the EV charging load and predicted charging load. The experiment results demonstrated that the MCCNN-TCN framework-based model outperformed the Artificial Neural Network (ANN), LSTM, CNN-LSTM, and TCN models in predicting the charging load of EVs and reduced the average absolute percentage error by up to 27.32%.

Li et al. [31] proposed a reinforcement learning-assisted deep learning framework to predict the charging load of EVs by training an LSTM deep learning model on charging power time series data. To address the uncertainty of EV charging behavior, the framework used a Markov decision process model and proximal policy optimization (PPO) algorithm to model the change in the LSTM model's component state. The authors also introduced an adaptive exploration PPO (AePPO) algorithm based on reinforcement learning to improve the PPO algorithm's adaptivity and avoid local optimization problems during the model training. The results showed that the LSTM-AePPO framework performed better than the LSTMPPO, Gradient Boosted Quantile Regression (GBQR), Quantile Regression (QR), and Quantile Regression Support Vector Machine (QRSVM) models. In [32], Jeon et al. proposed a hybrid model for predicting EV charging demand that combines traditional time series forecasting models and machine learning techniques, including dynamic harmonic regression (DHR), seasonal and trend decomposition, Bayesian structural time series (BSTS), random forest, extreme gradient boosting, and a stacked integrated learning architecture. Using the EV charging data set from the Korean Environmental Protection Agency, the authors compared the forecasting performance of the models trained with the new framework (Stack.XGBoost and Stack.GLM) and traditional time series statistical models (DHR, and BSTS) and showed that the dual-type framework can effectively improve prediction accuracy.

Van Kriekinge et al. [33] proposed an enhanced deep learning prediction system framework to predict EV charging demand for the succeeding day by integrating temperature and rainfall factors and generating time-dependent characteristic factors through data-processing techniques. The authors used an aggregated data set created via data engineering and trained three enhanced LSTM models, namely, LSTM-B, LSTM-C, and LSTM-W, using different combinations of EV charging characteristics, time-related factors, and temperature and rainfall characteristics. The authors used the charging data of a small hospital EV fleet to compare the accuracy of the three models and found that the LSTM-W model significantly outperformed the other models and reduced the mean absolute error (MAE) by 28.8% and root-mean-square error (RMSE) by 19.22%. Kim et al. [34] compared five machine learning and deep learning models for predicting EV charging, including Autoregressive Moving Average model (ARMA), Exponential smoothing state space model with Box-Cox transformation, ARMA errors, Trend and Seasonal components (TBATS), ARIMA, ANN, and LSTM models. The authors collected EV charging data from different areas in South Korea, from 2018 to 2019 and analyzed the data at macro and micro geographic regional scales. The authors found that charging capacity historical data are critical for predicting future charging demand and suggested that the further analysis, processing, and transformation of historical data can increase the polymorphism of data sets used for training models.

In [35], Zhou et al. presented a framework that combines Bayesian probability theory with Long Short-Term Memory (LSTM) networks to address both stochastic uncertainty—such as that arising from EV user behavior and environmental factors—and model uncertainty, which can result from varying model parameters and structures. This probabilistic deep learning approach is particularly valuable in scenarios where traditional methods fail to accurately capture the full predictive distribution at each time step. The authors propose a two-stage process: first, data preprocessing, which involves compiling temporal power consumption series and identifying relevant predictors, and second, forecasting using a Bayesian LSTM model. By placing a prior distribution on the weights and biases of the LSTM network, variational inference is employed to approximate the posterior distribution, effectively capturing the uncertainty in the forecast. The results demonstrate significant improvements in prediction accuracy, as evidenced by notable reductions in error metrics.

The study in [36] introduces a multi-step short-term EV charging load forecasting model, aimed at predicting charging loads at public EV stations using historical data. Unlike many existing models that focus on single-step predictions, this paper presents a robust framework called the DTCformer-BCMG model. The DTCformer-BCMG model integrates several advanced techniques, including temporal feature extraction, Discrete Cosine Transform (DCT)-based frequency enhancement, and the Bayesian-CNN-MHSA-GRU (BCMG) error correction block. These innovations address key challenges related to load sparsity, periodicity, and prediction accuracy.

Mohammad et al. [37] proposed an innovative architecture that combines encoder-decoder structures with ConvLSTM-based encoders and LSTM-based decoders, aimed at addressing both short- and long-term energy demand forecasting. This model specifically tackles the challenge of extracting both temporal and spatial information, which is particularly critical for forecasting demand at Electric Vehicle Charging Stations (EVCS). The authors evaluated their model using datasets from four cities in the UK and the USA, demonstrating its generalizability and scalability. By comparing their results to traditional machine learning and deep learning methods, the authors reported a significant improvement in prediction accuracy, with the model achieving up to a 33% reduction in RMSE.

In [38], Danish et al. addressed the increasing demand for EV charging and the resulting imbalanced load on distribution networks by proposing the Block-FeDL framework. This framework integrates Federated Learning (FL) with Blockchain technology to improve load forecasting and protect user privacy. Instead of a centralized server, Blockchain technology ensures secure, verifiable data exchange in a decentralized system. Federated Learning enables diverse EV charging data collection while maintaining user privacy. The model is updated securely through smart contracts on a decentralized network, with results showing significant improvements in forecasting accuracy.

The paper [39] introduces an advanced model, attention-SLSTM, which combines Stacked Long Short-Term Memory (SLSTM) with an attention mechanism to improve the accuracy of EV charging demand predictions. By utilizing stacked LSTM layers, the model enhances the extraction of features in time series forecasting. The attention mechanism further strengthens the model's ability to prioritize critical information within input sequences, allowing it to focus on the most relevant historical data. This leads to better interpretability and predictive performance by ensuring that important temporal dependencies are effectively captured.

In contrast to the aforementioned studies, our proposed method uniquely integrates queuing network models and Bayesian networks to evaluate the reliability of EV charging piles, taking into account both meteorological factors and charging pile failures. This integration offers a more holistic and comprehensive approach to understanding and predicting EV charging demand. Additionally, we introduce a HBNDL system architecture that merges queuing networks, Bayesian networks, and deep neural networks to create a robust model-training framework. This innovative framework enables the training of deep learning models across short-, medium-, and long-term scenarios, significantly enhancing prediction accuracy and adaptability. Our approach also stands out by utilizing extensive transaction data from Palo Alto, California, over a decade, providing a rich dataset for big data analytics to examine the impact of climatic factors on EV charging demand. The following sections detail the original methodology proposed in this study.

## 3 Materials and methods

**Figure 1:**
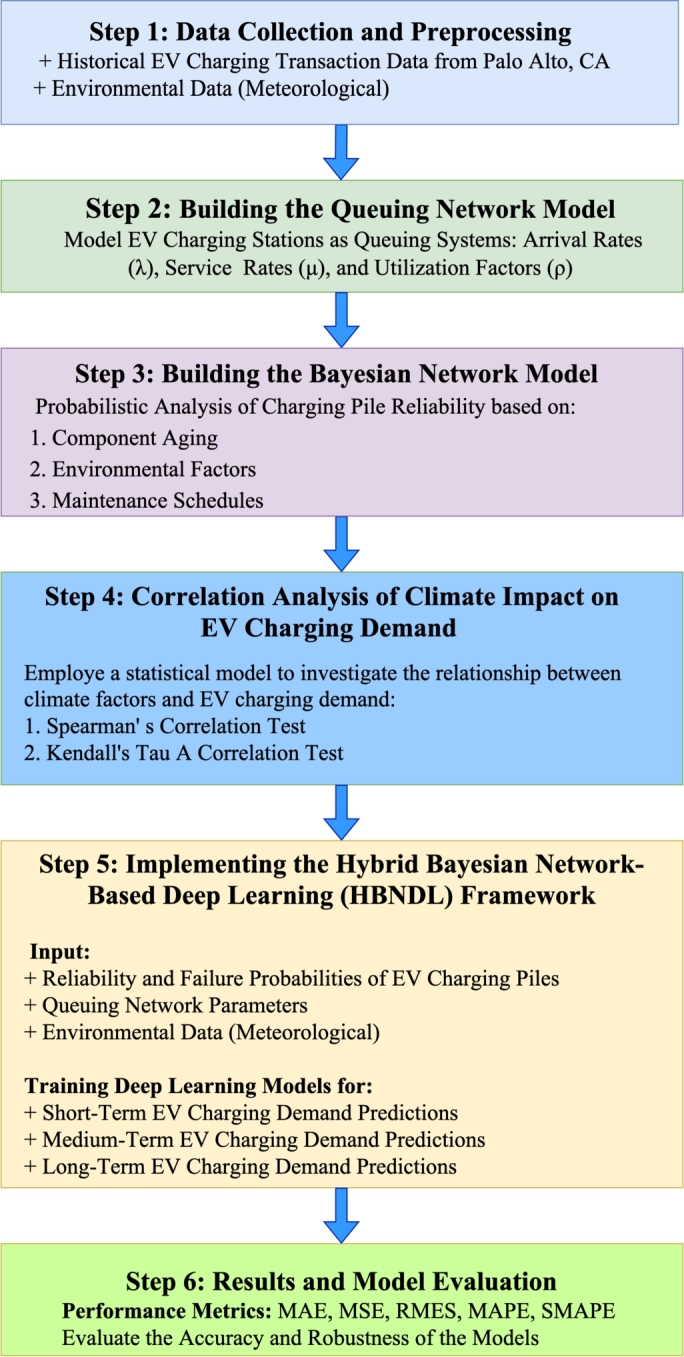
Overall research method flow chart.

This study presents a hybrid model that combines queuing network and Bayesian network analyses to assess the reliability of electric vehicle charging stations. The hybrid model incorporates environmental climate conditions and component aging as key factors. In the queuing network component, we utilize queuing theory to model both the waiting and service times of electric vehicle charging stations. The aim is to analyze queue length and system utilization, providing insight into the effectiveness of the charging infrastructure. In the Bayesian network component, we establish probabilistic relationships among various factors influencing the reliability of charging stations. Factors such as component aging, environmental conditions, and maintenance activities are integrated into the Bayesian network analysis. Fig. 1 illustrates the essential steps in the research design methodology. Each step is detailed as follows:

**Step 1: Data Collection and Preprocessing**. The first phase involves gathering historical transaction data from electric vehicle (EV) charging stations in Palo Alto, California [40]. This dataset includes charging times, charging energy, total duration, and other relevant station data. Additionally, environmental factors such as temperature, humidity, and precipitation are collected to assess their impact on charging demand. The data undergoes preprocessing to ensure cleanliness, normalization, and readiness for analysis. Any missing values are addressed, and the data is transformed into a format suitable for both queuing theory and Bayesian network analysis.

**Step 2: Building the Queuing Network Model**. The second step focuses on constructing a queuing network model to represent the behavior of charging stations as queuing systems. Key parameters, including the arrival rate (*λ*), service rate (*μ*), and utilization factor (*ρ*), are derived from the transaction data. The outputs of the queuing network model, such as the number of EVs in the system (*L*), the number of EVs waiting in the queue ($L_{q}$), and wait times (*W*), feed into both the Bayesian network model and the deep learning framework.

**Step 3: Building the Bayesian Network Model**. In this step, a Bayesian network is developed to calculate the reliability and failure probability of each charging station. The network integrates several probabilistic factors, including component aging, environmental conditions, and maintenance schedules. The key outputs of the Bayesian network model are the reliability and failure probabilities of the charging piles.

**Step 4: Correlation Analysis of Climate Impact on EV Charging Demand**. In this step, we analyze the correlation between environmental factors and EV charging demand to understand how weather patterns influence charging behavior. By examining historical climate data such as temperature, humidity, precipitation, and wind speed, alongside EV charging records, we identify patterns and trends in how these variables affect the demand for charging infrastructure. This correlation analysis helps refine the prediction model by incorporating environmental dynamics that may impact charging pile usage, system reliability, and charging demand fluctuations. The results of this analysis provide valuable insights for adapting the charging infrastructure to changing weather conditions and further enhance the predictive capabilities of the hybrid Bayesian network-based deep learning framework.

**Step 5: Implementing the Hybrid Bayesian Network-Based Deep Learning (HBNDL) Framework**. This step involves integrating the outputs from both the Bayesian and queuing network models into a deep learning framework. The HBNDL framework takes the following inputs: (i) the reliability and failure probabilities generated by the Bayesian network; (ii) queuing network parameters such as L, Lq, and W; and (iii) environmental data including temperature, humidity, and precipitation. Using these inputs, deep learning models such as LSTM and CNN are trained to predict short-, medium-, and long-term EV charging demand. This hybrid approach provides accurate and robust predictions by accounting for both operational and external factors like weather.

**Step 6: Results and Model Evaluation**. Once the models are trained, the performance of the hybrid framework is evaluated using key metrics such as Mean Absolute Error (MAE) and Root Mean Square Error (RMSE). These metrics are applied to assess how well the model performs across different time horizons and environmental conditions. The evaluation phase ensures that the model is robust, adaptable to real-world scenarios, and suitable for planning EV infrastructure.

### 3.1 Mathematical formulation for queuing network component

The symbols and their corresponding meanings in the hybrid model are presented in Table 1. The hybrid model integrates queuing theory to assess the dynamics of charging station usage and utilizes Bayesian networks to model the probabilistic relationships among various factors influencing the reliability of charging piles. This reliability metric is subsequently incorporated into a deep learning model to forecast EV charging demand. By combining deterministic and probabilistic methods, the hybrid model delivers robust and accurate predictions. The queuing network simulates the flow of electric vehicles at charging stations, calculating key performance indicators such as utilization rates, waiting times, and queue lengths. It employs the M/M/1 queuing system (Markovian arrivals, Markovian service times, single server) for its analysis.

**Table 1:** Symbols and definitions in hybrid Bayesian network model.

Symbols	Definitions
*λ*	Arrival rate (number of charging events per day)
*μ*	Service rate (number of charging events completed per day)
*L*	Average number of EVs in the system (including those being charged and waiting)
*L*_*q*_	Average number of EVs waiting in the queue
*W*_*t*_	Average time an EV spends in the system
*W*_*q*_	Average time an EV spends waiting in the queue
*ρ*	Traffic intensity (ratio of the arrival rate to the service rate)
*x*_*ev*_	EV charging infrastructure components
*λ*_1_	The scale parameter
*k*_1_	The shape parameter
*T*	Temperature
*H*	Humidity
*W*	Wind speed
*P*_*r*_	Precipitation

**Figure 2:**
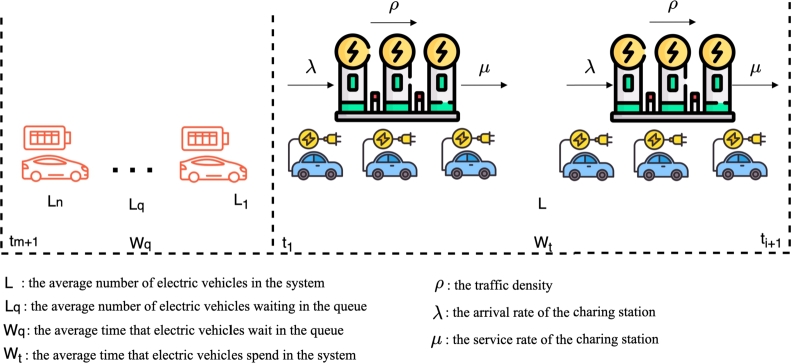
Queuing network diagram of the electric vehicle charging service system.

Within this model, we utilize a queuing network to model the waiting and service times of electric vehicle charging stations. Fig. 2 depicts a queuing network diagram of the electric vehicle charging service system. The traffic intensity *ρ* is defined as the ratio of the arrival rate *λ* of the charging station to the service rate *μ*, and can be expressed by Eq. (1). The average number of electric vehicles in the system is denoted by *L*, the average number of electric vehicles waiting in the queue is denoted by $L_{q}$, the average time that electric vehicles spend in the system is denoted by $W_{t}$, and the average time that electric vehicles wait in the queue is denoted by $W_{q}$. These values can be expressed by Eqs (2) to (5), respectively.

(1) $$\rho =\frac{\lambda }{\mu }$$

(2) $$L=\frac{\lambda }{\mu -\lambda }$$

(3) $$L_{q}=\frac{\lambda^{2}}{\mu (\mu -\lambda )}$$

(4) $$W_{t}=\frac{1}{\mu -\lambda }$$

(5) $$W_{q}=\frac{\lambda }{\mu (\mu -\lambda )}$$

### 3.2 Mathematical formulation for Bayesian network component

**Figure 3:**
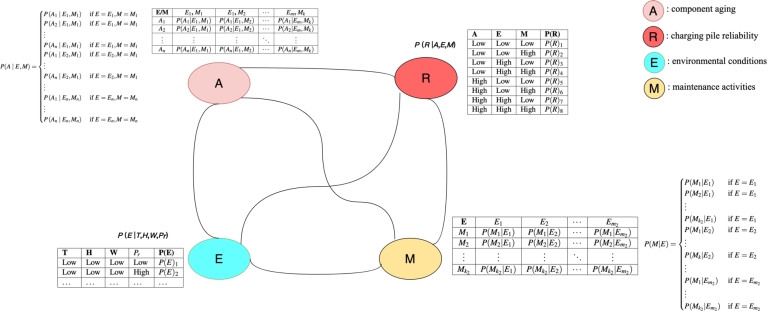
Bayesian network diagram of the electric vehicle charging service system.

We employ Bayesian networks to model the probabilistic relationships among various components of EV charging infrastructure. In this model, we integrate factors such as the aging of charging infrastructure components, environmental conditions (climate), and maintenance activities. The Bayesian network developed in this study includes four key nodes: electric vehicle charging pile reliability (R), component aging (A), environmental conditions (E), and maintenance activities (M). The graphical representation is illustrated in Fig. 3, aiding in the identification of critical factors influencing the reliability of electric vehicle charging piles and the optimization of maintenance strategies.

(1) Electric vehicle charging pile reliability (R): This node represents the reliability of electric vehicle charging piles, which is influenced by various factors including component aging (A), environmental conditions (E), and maintenance activities (M). R is a function of A, E, and M. The conditional probability distributions (CPDs) of R denoted by P(R|A,E,M) are presented in Table 2. As components age, the reliability of the charging pile may decrease, leading to a higher probability of failure (R). Environmental factors such as temperature, humidity, wind speed, and precipitation also impact the reliability of charging piles, with extreme weather conditions potentially reducing reliability. Maintenance activities, including regular inspections, repairs, and replacements, play a crucial role in ensuring the reliability of charging infrastructure.

**Table 2:** The conditional probability distributions of electric vehicle charging pile reliability (R).

A	E	M	P(R)
Low	Low	Low	*P*(*R*)_1_
Low	Low	High	*P*(*R*)_2_
Low	High	Low	*P*(*R*)_3_
Low	High	High	*P*(*R*)_4_
High	Low	Low	*P*(*R*)_5_
High	Low	High	*P*(*R*)_6_
High	High	Low	*P*(*R*)_7_
High	High	High	*P*(*R*)_8_

(2) Component aging (A): This node represents the aging of electric vehicle charging infrastructure components. The aging of components (A) directly affects the reliability of electric vehicle charging piles (R). As components age, their reliability may decline, increasing the likelihood of failure. Aging components may require more frequent maintenance to sustain reliability. Aging is modeled using a Weibull distribution, as expressed in Eq. (6), where $x_{ev}$ represents the age of the EV charging infrastructure component, $\lambda_{1}$ is the scale parameter, and $k_{1}$ is the shape parameter.

(6) $$f(x_{ev};\lambda_{1},k_{1})=\frac{k_{1}}{\lambda_{1}}{\left(\frac{x_{ev}}{\lambda_{1}}\right)}^{k_{1}-1}e^{-{\left(x_{ev}/\lambda_{1}\right)}^{k_{1}}}$$

The CPD for component aging (A) describes the probability distribution of aging states conditioned on environmental conditions and maintenance activities. Let *n* be the number of aging states, *m* be the number of environmental conditions, and *k* be the number of maintenance activities. The conditional probability P(A|E,M) can be represented as an n×m×k matrix, as expressed in Eq. (7), where $E_{i}$ represents the *i*-th environmental condition, $M_{j}$ represents the *j*-th maintenance activity, and *k* represents the *k*-th aging state. Table 3 provides an example of the CPD for component aging (A).

(7) $$P(A|E,M)=\left\{ \begin{array}{cc} P\left(A_{1}|E_{1},M_{1}\right) & \text{ if }E=E_{1},M=M_{1}\\ P\left(A_{2}|E_{1},M_{1}\right) & \text{ if }E=E_{1},M=M_{1}\\ \vdots & \\ P\left(A_{n}|E_{1},M_{1}\right) & \text{ if }E=E_{1},M=M_{1}\\ P\left(A_{1}|E_{2},M_{1}\right) & \text{ if }E=E_{2},M=M_{1}\\ \vdots & \\ P\left(A_{n}|E_{2},M_{1}\right) & \text{ if }E=E_{2},M=M_{1}\\ \vdots & \\ P\left(A_{1}|E_{n},M_{n}\right) & \text{ if }E=E_{n},M=M_{n}\\ \vdots & \\ P\left(A_{n}|E_{n},M_{n}\right) & \text{ if }E=E_{n},M=M_{n} \end{array}\right.$$

**Table 3:** The conditional probability distributions of component aging (A).

E/M	*E*_1_, *M*_1_	*E*_1_, *M*_2_	⋯	*E*_*m*_, *M*_*k*_
*A*_1_	*P*(*A*_1_|*E*_1_,*M*_1_)	*P*(*A*_1_|*E*_1_,*M*_2_)	⋯	*P*(*A*_1_|*E*_*m*_,*M*_*k*_)
*A*_2_	*P*(*A*_2_|*E*_1_,*M*_1_)	*P*(*A*_2_|*E*_1_,*M*_2_)	⋯	*P*(*A*_2_|*E*_*m*_,*M*_*k*_)
⋮	⋮	⋮	⋱	⋮
*A*_*n*_	*P*(*A*_*n*_|*E*_1_,*M*_1_)	*P*(*A*_*n*_|*E*_1_,*M*_2_)	⋯	*P*(*A*_*n*_|*E*_*m*_,*M*_*k*_)

(3) Environmental conditions (E): This node represents environmental factors such as temperature (*T*), humidity (*H*), wind speed (*W*), and precipitation ($P_{r}$). The impact of each environmental factor on reliability can be modeled using conditional probability distributions, denoted by $P(E|T,H,W,P_{r})$, as illustrated in Table 4. Environmental conditions (E) significantly influence the reliability (R) of electric vehicle charging piles. High temperatures, extreme humidity, strong winds, and heavy precipitation can diminish the reliability of charging infrastructure. Extreme weather conditions can accelerate component aging (A) and elevate the risk of failure (R). Additionally, environmental conditions may affect the frequency and type of maintenance activities (M) required.

**Table 4:** The conditional probability distributions of environmental conditions (E).

T	H	W	*P*_*r*_	P(E)
Low	Low	Low	Low	*P*(*E*)_1_
Low	Low	Low	High	*P*(*E*)_2_
…	…	…	…	…
High	High	High	High	*P*(*E*)_*i*_

(4) Maintenance activities (M): This node represents maintenance tasks such as inspection, repair, and replacement. Maintenance activities (M) directly impact the reliability (R) of electric vehicle charging piles. Regular inspections, timely repairs, and proactive replacements are essential for maintaining or improving reliability. The CPD for Maintenance Activities (M) describes the probability distribution of maintenance activities conditioned on environmental conditions. Let $k_{2}$ be the number of maintenance activities and $m_{2}$ be the number of environmental conditions. The conditional probability P(M|E) can be represented as a k2×m2 matrix and mathematically expressed in Eq. (8). Table 5 illustrates the CPD of Maintenance Activities (M), where $E_{i}$ represents the *i*-th environmental condition and $M_{j}$ represents the *j*-th maintenance activity. This CPD table depicts the probability distribution of maintenance activities conditioned on different environmental conditions.

**Table 5:** The conditional probability distribution for maintenance activities (M).

E	*E*_1_	*E*_2_	⋯	$E_{m_{2}}$
*M*_1_	*P*(*M*_1_|*E*_1_)	*P*(*M*_1_|*E*_2_)	⋯	$P(M_{1}|E_{m_{2}})$
*M*_2_	*P*(*M*_2_|*E*_1_)	*P*(*M*_2_|*E*_2_)	⋯	$P(M_{2}|E_{m_{2}})$
⋮	⋮	⋮	⋱	⋮
$M_{k_{2}}$	$P(M_{k_{2}}|E_{1})$	$P(M_{k_{2}}|E_{2})$	⋯	$P(M_{k_{2}}|E_{m_{2}})$

(8) $$P(M|E)=\left\{ \begin{array}{cc} P(M_{1}|E_{1}) & \text{if }E=E_{1}\\ P(M_{2}|E_{1}) & \text{if }E=E_{1}\\ \vdots \\ P(M_{k_{2}}|E_{1}) & \text{if }E=E_{1}\\ P(M_{1}|E_{2}) & \text{if }E=E_{2}\\ \vdots \\ P(M_{k}|E_{2}) & \text{if }E=E_{2}\\ \vdots \\ P(M_{1}|E_{m_{2}}) & \text{if }E=E_{m_{2}}\\ \vdots \\ P(M_{k_{2}}|E_{m_{2}}) & \text{if }E=E_{m_{2}} \end{array}\right.$$

### 3.3 Mathematical formulation for hybrid Bayesian model

The Bayesian network captures the probabilistic dependencies between key variables, including charging pile reliability, environmental conditions, component aging, and maintenance activities. It estimates the conditional probability distributions (CPDs) for the following variables: Conditional Probability of Reliability P(R|A,E,M), Component Aging P(A|E,M), and Environmental Conditions P(E|T,H,W,Pr). These conditional probabilities are aggregated within the Bayesian network to compute the overall reliability of each charging pile, which serves as an input for the deep learning model to predict future demand. The charging pile's reliability P(R) is defined using Eq. (9), which integrates the impacts of aging, environmental factors, and maintenance into a singular probability.

(9) P(R)=P(R|A,E,M)⋅P(A|E,M)⋅P(E|T,H,W,Pr)

The combined reliability of EV charging piles is calculated by integrating the queuing network component with the Bayesian network component, as expressed in Eq. (10), where P(R|A,E,M) represents the conditional probability of EV Charging Pile Reliability given Component Aging (A), Environmental Conditions (E), and Maintenance Activities (M) from the Bayesian network. P(A) is the probability of Component Aging (A) obtained from the CPD for Component Aging. Similarly, P(E) and P(M) represent the probabilities of Environmental Conditions (E) and Maintenance Activities (M) obtained from their respective CPDs. The queuing network component, $g(L,L_{q},W_{t},W_{q})$, incorporates queuing parameters such as the average number of EVs in the system (*L*), the average number of EVs waiting in the queue (*Lq*), the average time an EV spends in the system ($W_{t}$), and the average time an EV spends waiting in the queue ($W_{q}$).

(10) $$R_{\text{combined }}=\sum_{R}P(R|A,E,M)\times P(A)\times P(E)\times P(M)\times g\left(L,L_{q},W_{t},W_{q}\right)$$

(11) $$P_{\text{failure}}=1-R_{\text{combined}}$$

The probability of EV charging pile failure ($P_{failure}$) is determined by the combined reliability model, calculated using Eq. (11). Our hybrid model integrates queuing networks with Bayesian networks to offer a comprehensive analysis of the reliability and performance of EV charging infrastructure. Queuing networks are employed to model the waiting time, service time, queue length, and system utilization of EV charging piles, while Bayesian networks model the probabilistic relationships among various factors influencing charging pile reliability, including component aging, environmental conditions, and maintenance activities. By combining these two components, the hybrid model provides a more accurate and comprehensive analysis of EV charging pile reliability. Furthermore, the charging pile failure rate calculated by the hybrid model is used as a parameter for training the deep learning model, significantly enhancing the accuracy of the deep learning model in predicting electric vehicle charging demand. This integration of advanced modeling techniques ensures not only the reliability and efficiency of EV charging infrastructure but also enhances the accuracy of predicting charging demand, thereby contributing to the sustainable development of electric mobility. Algorithm 1 presents pseudocode that employs the queuing network model to estimate the usage of charging stations. Algorithm 2 integrates both the queuing network model and the Bayesian network model to calculate the probabilities of reliability and failure for the charging piles.

**Algorithm 1:**
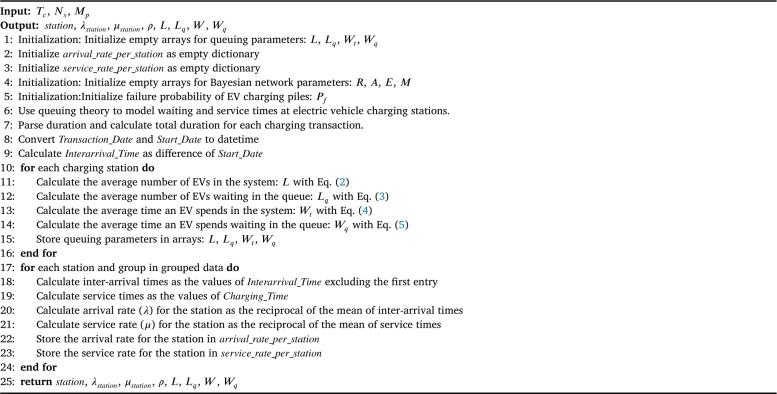
Assessing electric vehicle charging station usage using a queuing network model.

**Algorithm 2:**
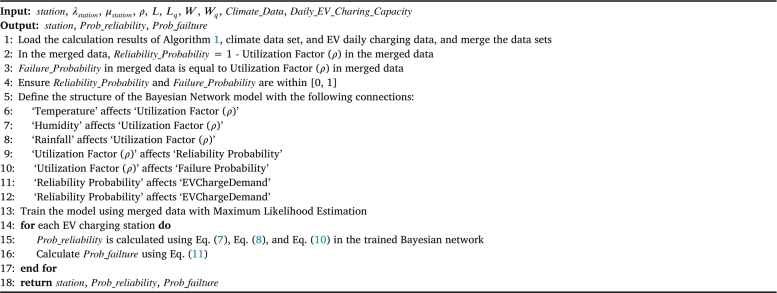
Calculation of reliability and failure probabilities for electric vehicle charging stations using an integrated queuing network and Bayesian network model.

The computed reliability P(R) from the Bayesian network is a key feature used by the deep learning model to predict EV charging demand over time. The deep learning model's forecast for future demand is given by Eq. (12). Where, $D_{t+k}$ represents the predicted charging demand at time t+k. $D_{t}$ is the historical charging demand. $E_{t}$ refers to environmental condition data. $P(R_{t})$ is the charging pile reliability at time *t*, derived from the Bayesian network. *θ* denotes the model parameters learned during training.

(12) $$D_{t+k}=f(D_{t},E_{t},P(R_{t}),\theta )$$

The model is trained by minimizing a loss function (L) given by Eq. (13). Where, $D_{i,t+k}$ is the actual charging demand for station *i*. ${\overset{ˆ}{D}}_{i,t+k}$ represents the predicted demand. *N* is the number of data points.

(13) $$L(\theta )=\frac{1}{N}\sum_{i=1}^{N}{\left(D_{i,t+k}-{\overset{ˆ}{D}}_{i,t+k}\right)}^{2}$$

This mathematical model offers a robust framework for analyzing the reliability and performance of electric vehicle charging infrastructure, enabling the optimization of system operation and maintenance. The hybrid model provides a comprehensive analysis of the reliability and performance of electric vehicle charging infrastructure, considering operational and environmental factors. By combining queuing theory with Bayesian networks, the hybrid model enhances the accuracy of predicting electric vehicle charging pile failures, consequently improving the accuracy of predicting electric vehicle charging capacity.

### 3.4 Correlation analysis of climate impact on EV charging demand

**Figure 4:**
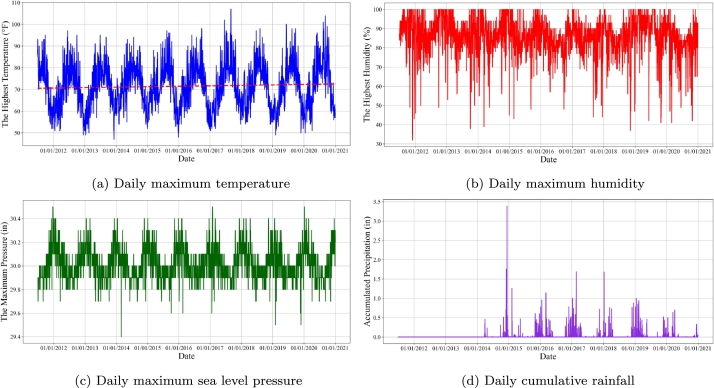
The historical climate changes in Palo Alto, from July 2011 to December 2020.

We collected the historical meteorological data of Palo Alto, from July 2011 to December 2020 from the professional meteorological website Weather Underground [41] to investigate the impact of extreme climate changes on EV charging demand. Fig. 4 displays the climate changes in Palo Alto, from July 2011 to December 2020, presenting the daily maximum temperature (Fig. 4(a)), daily maximum humidity (Fig. 4(b)), daily maximum sea level pressure (Fig. 4(c)), and daily cumulative rainfall (Fig. 4(d)). The red trend line in Fig. 4(a) reveals a gradual upward trend in the daily maximum temperature in Palo Alto, which can be attributed to global warming. Notably, some days experienced abnormally high temperatures, such as September 1 and 2, 2017, when the maximum temperature reached 107 °F (41.67 °C). Furthermore, the accumulated rainfall on December 12, 2014 was recorded at 3.39 inches (86.784 mm), which reached the level of heavy rain, as shown in Fig. 4(d).

We employed a statistical model in this study to investigate the relationship between climate factors and EV charging demand. Although Pearson's correlation test is the most commonly used method, it measures only the strength of the linear relationship between two sets of data and requires certain assumptions, such as interval or ratio levels, linear correlation, and bivariate normal distribution. Such assumptions were not met by the climate factors and EV charging demand data; thus, we conducted Spearman's and Kendall's rank correlation tests, with Kendall's tau-a correlation test being the preferred method. In a correlation analysis, the two-tailed tested p-value is employed in the significance test, with the predetermined significance level (*α*) set to 0.05. The p-value serves as a threshold for determining the statistical significance of the correlation and evaluates whether the observed correlation is attributable to chance or signifies a genuine relationship between the variables.

The results presented in Table 6 established a correlation between EV charging capacity and various weather factors. The obtained p-values indicated that some correlations were statistically significant (p<0.05), whereas others were not. The weather factors that had a statistically significant positive correlation with EV charging capacity included the average temperature, minimum temperature, maximum dew point temperature, average dew point temperature, minimum dew point temperature, minimum humidity, maximum wind speed, average wind speed, minimum wind speed, and accumulated precipitation. The results suggested that an increase in such weather factors corresponded to an increase in the total amount of EV charging. However, maximum humidity exhibited a statistically significant negative correlation with charging demand, which implied that an increase in humidity was associated with a decrease in the total amount of EV charging. By contrast, no statistically significant correlation existed between maximum temperature, average humidity, air pressure, and charging capacity. The results of Kendall's test are consistent with those of Spearman's test, which showed that climate variables such as temperature, dew point, wind speed, and precipitation can significantly impact the EV charging amount. Therefore, such weather factors can be used as features in the training data set to train a model for predicting EV charging demand and improve the model prediction accuracy.

**Table 6:** Correlation test between EV charging demand and climatic variables.

	Spearman's Correlation Coefficient		Kendall's Tau A Correlation Coefficient	
Climatic Variables	*ρ*	p-value	*τ*	p-value
Temperature_High	0.016	0.352	0.011	0.320
Temperature_Avg	0.057	**0.001**^⁎^	0.038	**0.001**^⁎^
Temperature_Low	0.063	**0.000**^⁎^	0.043	**0.000**^⁎^
Dew_Point_High	0.052	**0.002**^⁎^	0.035	**0.002**^⁎^
Dew_Point_Avg	0.060	**0.000**^⁎^	0.039	**0.001**^⁎^
Dew_Point_Low	0.068	**0.000**^⁎^	0.046	**0.000**^⁎^
Humidity_High	-0.088	**0.000**^⁎^	-0.059	**0.000**^⁎^
Humidity_Avg	-0.002	0.913	-0.001	0.946
Humidity_Low	0.082	**0.000**^⁎^	0.056	**0.000**^⁎^
Speed_High	0.062	**0.000**^⁎^	0.043	**0.000**^⁎^
Speed_Avg	0.110	**0.000**^⁎^	0.074	**0.000**^⁎^
Speed_Low	0.042	**0.013**^⁎^	0.034	**0.013**^⁎^
Pressure_High	0.003	0.860	0.002	0.867
Pressure_Avg	-0.014	0.426	-0.010	0.423
Pressure_Low	-0.006	0.703	-0.005	0.685
Precip.Accum._Sum	0.204	**0.000**^⁎^	0.163	**0.000**^⁎^

Note: * denote significance at the 5% level.

### 3.5 HBNDL system architecture

**Figure 5:**
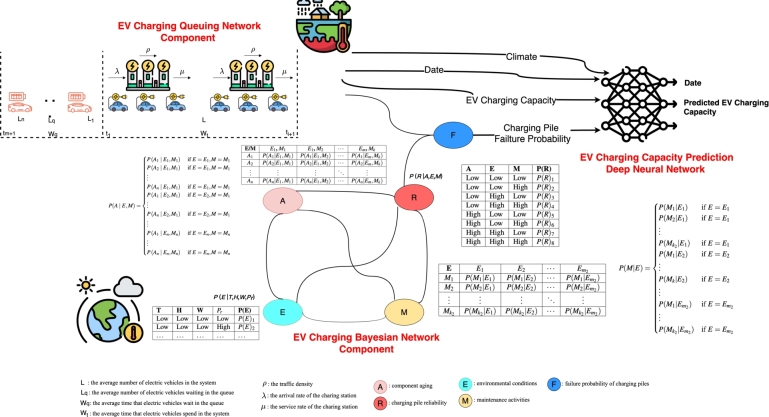
System architecture diagram for predicting electric vehicle charging capacity based on hybrid Bayesian network and deep learning neural network.

The HBNDL system architecture proposed in this study is based on a hybrid Bayesian network model and a deep learning model, as illustrated in Fig. 5. The system integrates data from various sources, including the EV charging network, climate data in the charging station's location, and other relevant factors. It comprises three main components: an EV charging queuing network modeling component, an EV charging Bayesian network modeling component, and an EV charging demand prediction deep neural network component.

The EV charging queuing network modeling component employs queuing theory to model waiting and service times at electric vehicle charging stations. Its primary objective is to analyze queue lengths and system utilization, providing insights into the effectiveness of the charging infrastructure. The EV charging Bayesian network modeling component establishes probabilistic relationships between various factors influencing the reliability of charging stations. Factors such as component aging, environmental conditions, and maintenance activities are incorporated into the Bayesian network analysis to calculate the failure probability of EV charging piles. The EV charging demand prediction deep neural network component utilizes time and historical data of electric vehicle charging capacity, along with the calculated failure probability of EV charging piles, this component trains various deep learning time series prediction models. These models enable short-term, medium-term, and long-term electric vehicle charging demand forecasts, thereby enhancing forecast accuracy.

The integration of Bayesian networks and deep learning in this study enhances the accuracy of predicting electric vehicle (EV) charging demand by combining the strengths of both approaches—Bayesian networks for probabilistic inference and deep learning for feature extraction and pattern recognition. Bayesian networks are graphical models used to represent variables and their probabilistic dependencies. In the context of EV charging demand forecasting, Bayesian networks model the relationships between key factors, including charging pile reliability, component aging, environmental conditions, and maintenance activities. Charging pile reliability represents the likelihood of the piles functioning, influenced by environmental conditions and component aging, while component aging reflects the wear and tear of components over time. Environmental conditions such as temperature, humidity, and precipitation affect EV charging patterns and infrastructure reliability, and maintenance activities account for planned or reactive maintenance that impacts the operational status of the charging piles. By using conditional probability distributions (CPDs), Bayesian networks calculate the failure probabilities of charging piles under various scenarios, which are then used as input features for the deep learning model. This probabilistic modeling helps capture system uncertainty—crucial for time series forecasting.

Once the failure probabilities are determined, deep learning is used to forecast EV charging demand over time. The Hybrid Bayesian Network-based Deep Learning (HBNDL) framework integrates these failure probabilities with other historical and environmental data, such as past charging demand, weather conditions, and time of day. The deep learning model employs Long Short-Term Memory (LSTM) networks, which are particularly suited for capturing temporal dependencies in time series data. In this process, the model learns how weather conditions and charging pile failure probabilities influence charging demand fluctuations over time, improving the accuracy of predictions.

The LSTM model takes historical charging demand, failure probabilities derived from the Bayesian network, and environmental conditions as input. The LSTM layers process this sequential data, capturing temporal patterns and dependencies. Finally, the model outputs the predicted future charging demand for different time horizons, accounting for short-, medium-, and long-term scenarios. By integrating failure probabilities, the model considers the probabilistic impact of infrastructure reliability, which enhances prediction accuracy.

The key to this integration lies in how the Bayesian network models the uncertainty of charging infrastructure reliability and how this uncertainty is incorporated into the deep learning model to improve time series forecasting. The failure probabilities from the Bayesian network enable the LSTM model to better account for external factors such as weather and component aging, which may cause disruptions in charging infrastructure and influence demand. Without this integration, the deep learning model would rely solely on historical demand and weather data. By combining these data points with probabilistic insights into system reliability, the model gains a deeper understanding of the factors influencing demand and becomes better equipped to forecast extreme scenarios, such as sudden spikes or drops in demand due to weather or equipment failures.

## 4 Experiments and results discussion

To verify the effectiveness of the proposed HBNDL framework in enhancing the accuracy of deep learning models for electric vehicle charging capacity prediction, we trained five representative models: MLP [42], Convolutional Neural Network (CNN) [43], LSTM [44], sequence-to-sequence with attention (Seq2Seq-Attention) [45], and Learning a Vector Representation of Time (Time2Vec) [46]. We conducted a comprehensive performance evaluation across three experimental scenarios to compare the predictive capabilities of models without the HBNDL framework against those with the HBNDL framework in multivariate time series prediction. The multivariate variables included electric vehicle charging demand, charging pile failure probability, and daily cumulative rainfall.

In this study, we used five essential model performance metrics to evaluate the performance of an improved time series deep learning model trained using the designed HBNDL framework. The metrics were the MAE, mean squared error (MSE), RMSE, MAPE, and symmetric mean absolute percentage error (SMAPE). The calculations of MAE, MSE, RMSE, MAPE, and SMAPE are illustrated in Equations (14) - (18), where $y_{i}$ represents the actual value, ${\overset{ˆ}{y}}_{i}$ represents the predicted value, and *n* is the number of samples.

(14) $$\mathrm{MAE}=\frac{1}{n}\sum_{i=1}^{n}\left|y_{i}-{\overset{ˆ}{y}}_{i}\right|$$

(15) $$\mathrm{MSE}=\frac{1}{n}\sum_{i=1}^{n}{\left(y_{i}-\overset{ˆ}{y_{i}}\right)}^{2}$$

(16) $$\mathrm{RMSE}=\sqrt{MSE}=\sqrt{\frac{1}{n}\sum_{i=1}^{n}{\left(y_{i}-\overset{ˆ}{y_{i}}\right)}^{2}}$$

(17) $$\mathrm{MAPE}=\frac{100\text{\%}}{n}\sum_{t=1}^{n}\left|\frac{y_{i}-\overset{ˆ}{y_{i}}}{y_{i}}\right|$$

(18) $$\mathrm{SMAPE}=\frac{100\text{\%}}{n}\sum_{i=1}^{n}\frac{\left|\overset{ˆ}{y_{i}}-y_{i}\right|}{\left(\left|y_{i}\right|+\left|\overset{ˆ}{y_{i}}\right|\right)/2}$$

### 4.1 Experiment 1: validation of the integrated Bayesian network and queuing network model for evaluating the reliability of electric vehicle charging piles

**Figure 6:**
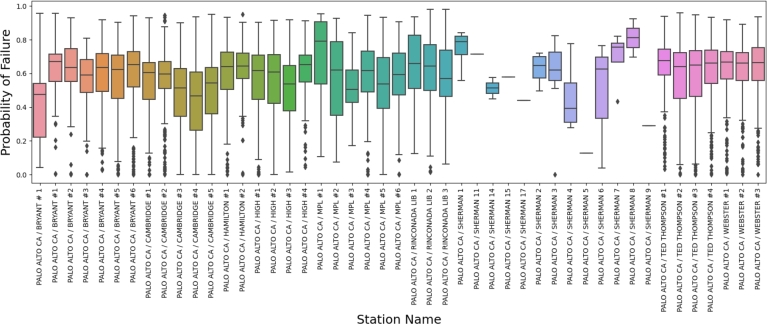
Distribution of failure probability of electric vehicle charging piles in Palo Alto in 2020 calculated using a hybrid Bayesian network model.

To validate the efficacy of the integrated Bayesian network and queuing network model for evaluating the reliability of electric vehicle charging pile proposed in this study, we conducted an analysis of the Palo Alto charging station's computation outcomes utilizing HBNDL. As illustrated in Fig. 6, the distribution displays the highest probability of failure across 47 charging stations in Palo Alto City during 2020. Out of the total 47 charging stations utilized in 2020, the aggregate probability of failure across all stations was 0.57. Notably, the charging station PALO ALTO CA/RINCONADA LIB 2 exhibited the highest probability of failure at 0.98, recorded on June 25, 2020. Conversely, the probability of malfunction for charging station PALO ALTO CA/WEBSTER #2 on June 29, 2020, was determined to be 0.

**Figure 7:**
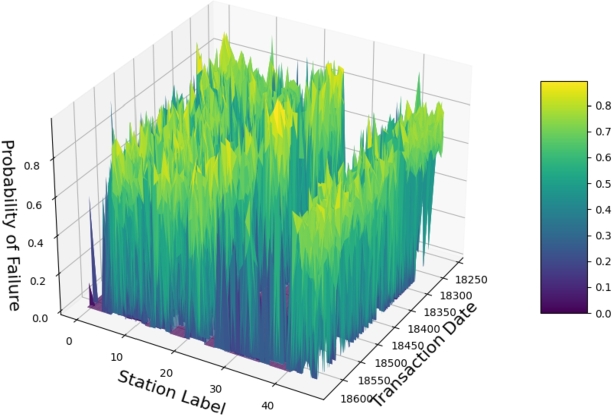
3D diagram of daily failure probability across 47 charging stations in Palo Alto city in 2020.

Fig. 7 depicts a 3D diagram illustrating the probability of daily failure across 47 charging stations in Palo Alto City throughout 2020. The x-axis represents the charging stations, the y-axis indicates the chronological order of transaction dates when the charging stations were in use, and the z-axis denotes the probability of failure for each charging station. Additionally, we conducted a detailed analysis of the methodology proposed in this study to investigate the influence of meteorological variables on the charging demand of electric vehicles.

**Figure 8:**
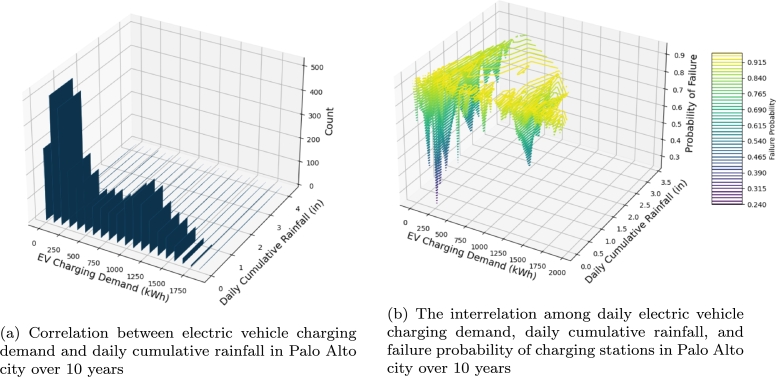
Analysis of climate factors and charging pile failure impact on electric vehicle charging demand in the HBNDL framework.

Fig. 8(a) depicts our examination of the correlation between electric vehicle charging demand in Palo Alto City and the city's daily cumulative rainfall over a span of 10 years. Our findings indicate a negative correlation between daily rainfall and electric vehicle charging demand. Specifically, we observed that higher levels of rainfall correspond to lower charging demand, and conversely, lower levels of rainfall are associated with increased charging demand. Therefore, the inclusion of daily rainfall as a feature in the HBNDL framework developed in this study for training deep learning prediction models is justified. Fig. 8(b) presents the outcomes of our analysis regarding the interrelation among daily electric vehicle charging demand, daily cumulative rainfall, and the failure probability of electric vehicle charging stations in Palo Alto City over a span of 10 years. It is evident that higher probabilities of charging station failure and increased daily rainfall correspond to diminished electric vehicle charging demand on that particular day. Notably, when the charging station failure probability exceeds 0.5 and daily cumulative rainfall surpasses 2.3 inches, the electric vehicle charging demand falls below 750 kWh. Consequently, the HBNDL framework advocated in this study amalgamates factors associated with electric vehicle charging station failures and meteorological conditions. Such integration facilitates the training of deep neural network models to unravel the intricate factors influencing electric vehicle charging capacity and enhances prediction accuracy by discerning underlying patterns.

### 4.2 Experiment 2: analysis of prediction accuracy over forecast steps within the HBNDL framework

This experiment aims to assess the predictive accuracy of the deep learning model trained within the HBNDL framework for forecasting EV charging demand at various future time intervals. The impact of the forecasting steps on the forecasting accuracy of a model is crucial for evaluating and comprehending the performance of time series forecasting models, particularly for EV charging demand prediction.

**Figure 9:**
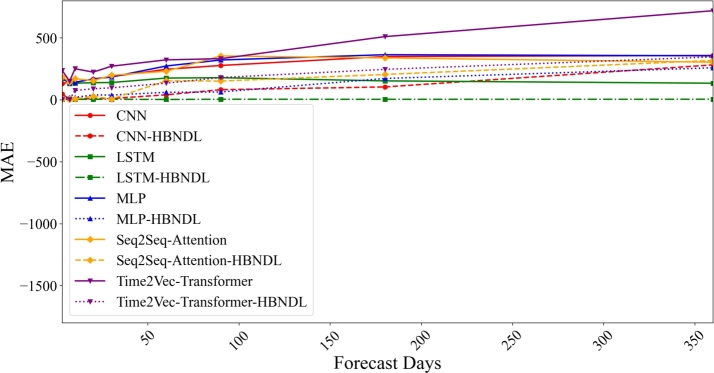
Comparison of the impact of forecasting steps on the MAE predicted by the models.

Fig. 9 illustrates the influence of the forecast horizon on the predictive accuracy of the models. Each line in the graph corresponds to a specific forecasting model. The x-axis represents the number of forecast steps, ranging from 3 days to 360 days, while the y-axis indicates the forecast error, with lower values signifying enhanced predictive accuracy. The solid lines depict the MAE of five native models for forecasting electric vehicle charging capacity, and the dotted lines represent the MAEs of the models trained within the HBNDL framework. The experimental findings reveal a substantial improvement in the accuracy of neural network models across various forecast horizons with the introduction of the HBNDL framework. Notably, all models trained using the HBNDL framework consistently exhibit significantly smaller prediction errors compared to the corresponding native models. For instance, compared with the LSTM model without the HBNDL framework, the LSTM-HBNDL model demonstrated a reduction in MAE by 98.23%. Furthermore, a comparison between the basic CNN model and the CNN-HBNDL model demonstrates a substantial reduction in MAE. These results underscore the pivotal role of HBNDL in enhancing model performance.

**Figure 10:**
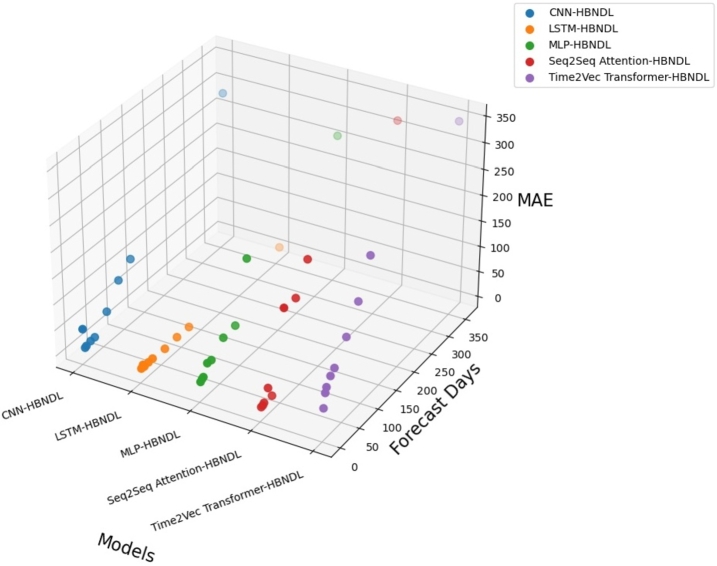
Comparison of MAE for predicting future daily charging volume for five models trained using the HBNDL framework.

Fig. 10 presents a comparison of the MAE for five models trained using the HBNDL framework to predict the daily charge amount for various future steps. It is evident from the figure that the LSTM-HBNDL model exhibits the lowest MAE, approximately 5.67, followed by the Seq2Seq Attention-HBNDL model, with a MAE of around 7.89. Conversely, the CNN-HBNDL model demonstrates the highest MAE, approximately 67.36.

**Figure 11:**
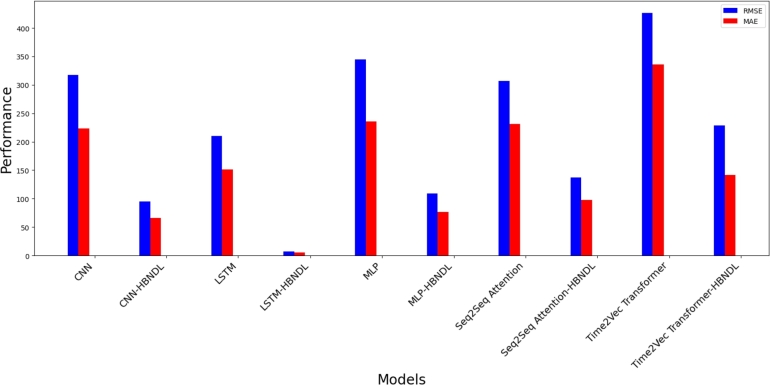
Comparison of RMSE and MAE for electric vehicle charging demand forecasting models with and without HBNDL framework training.

**Figure 12:**
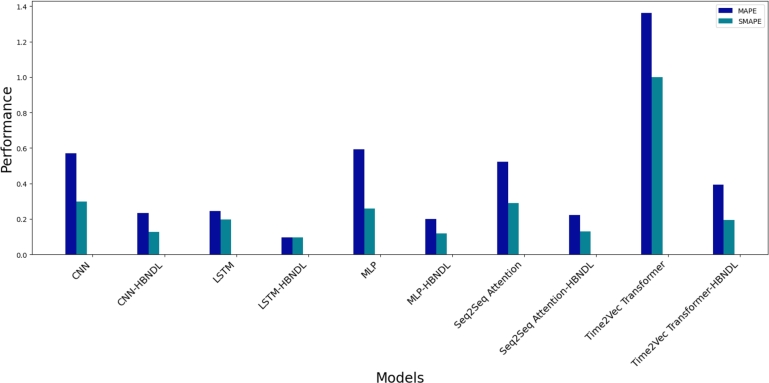
Comparison of MAPE and SMAPE for electric vehicle charging demand forecasting models with and without HBNDL framework training.

Figure 11, Figure 12 provide further insight into the comparative prediction performance of all models. Fig. 11 delineates a comparison of the RMSE and MAE predicted by each model, while Fig. 12 contrasts the MAPE and SMAPE predicted by the models. Our experimental findings highlight that the LSTM-HBNDL model, trained within the HBNDL framework, exhibits superior overall performance. The LSTM-HBNDL model demonstrates enhanced proficiency in forecasting short-, medium-, and long-term electric vehicle charging demands, surpassing other models, including LSTM models not trained within the HBNDL framework, in accuracy and fulfillment of quantitative requirements.

The HBNDL framework showcases its effectiveness across various model architectures, including CNN, LSTM, MLP, Seq2Seq Attention, and Time2Vec Transformer. This versatility highlights the framework's capacity to enhance a diverse set of forecasting models. Importantly, the improvements brought about by HBNDL are more pronounced as the forecast horizon extends. Notably, in the Time2Vec Transformer-HBNDL model, the MAE experiences a substantial reduction, particularly for long-term predictions (e.g., 180 and 360 days), underscoring the framework's capability to capture and integrate complex contextual information that becomes increasingly critical in longer-term forecasts.

### 4.3 Experiment 3: analysis of prediction accuracy over training epochs within the HBNDL framework

The purpose of conducting an experiment analysis on the impact of model training epochs on the model prediction accuracy was to identify the optimal number of training epochs necessary to achieve the best model performance. In the field of machine learning and deep learning, the training epoch refers to the number of times a model processes the entire data set during the training. During the initial stages of the training, the model will tend to overfit the training data, which can lead to high training accuracy but low validation accuracy. As the training progresses, the model will improve its ability to generalize new and unseen data, which will result in enhanced validation accuracy. However, beyond a certain point, the model may begin to overfit the validation data, which will lead to reduced validation accuracy. Thus, determining the optimal number of training epochs is crucial for optimizing the model's performance. The experiment analysis involves training a model with different numbers of epochs and evaluating the resulting prediction accuracy using an independent validation set within the HBNDL framework.

**Figure 13:**
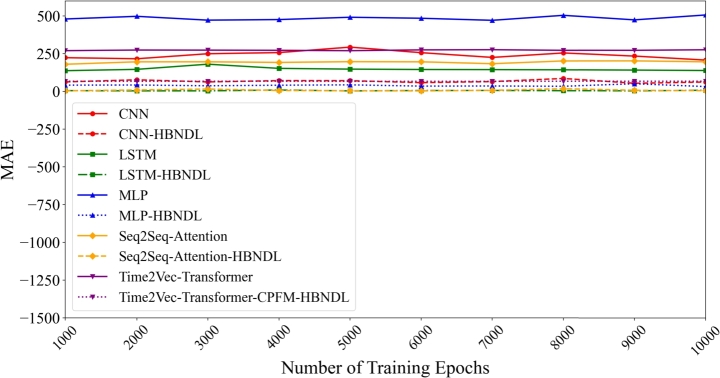
Comparison of the impact of the number of training epochs on the MAE predicted by the models.

Fig. 13 illustrates the influence of training epochs on the forecast accuracy of the models. The x-axis denotes the number of training epochs, ranging from 1000 to 10000, while the y-axis represents the forecast error. The experimental results clearly demonstrate the substantial impact of incorporating the HBNDL framework in enhancing the accuracy of all models. Starting with the performance of CNN-based models, it is evident that the CNN-HBNDL model consistently surpasses the basic CNN model. This superiority is observable across all epochs, marked by a significant reduction in MAE achieved by the HBNDL-enhanced model. Notably, the CNN-HBNDL model exhibits improved accuracy as the number of training epochs increases, indicating its capacity to adapt and learn from the data. Similar trends are discernible in the context of LSTM-based models. The LSTM-HBNDL model consistently presents substantially lower MAE values compared to the standard LSTM model. This pattern remains consistent across diverse training epochs, underscoring the effectiveness of the HBNDL framework in augmenting LSTM-based forecasting. In the case of MLP models, a consistent reduction in MAE is observed with the inclusion of the HBNDL framework, illustrating its constructive impact on MLP-based forecasting models. The MLP-HBNDL model consistently outperforms the basic MLP model across all epochs. Moreover, the Seq2Seq Attention and Time2Vec Transformer models demonstrate the efficacy of the HBNDL framework. Both the Seq2Seq Attention-HBNDL and Time2Vec Transformer-HBNDL models consistently achieve lower MAE values than their respective basic models.

**Figure 14:**
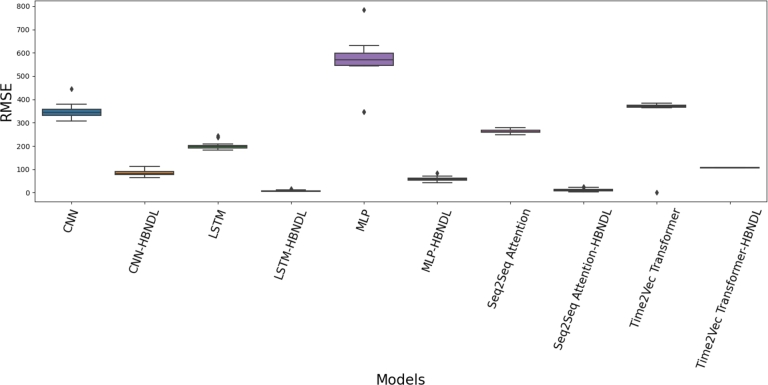
Comparison of the RMSE of the models over training epochs.

**Figure 15:**
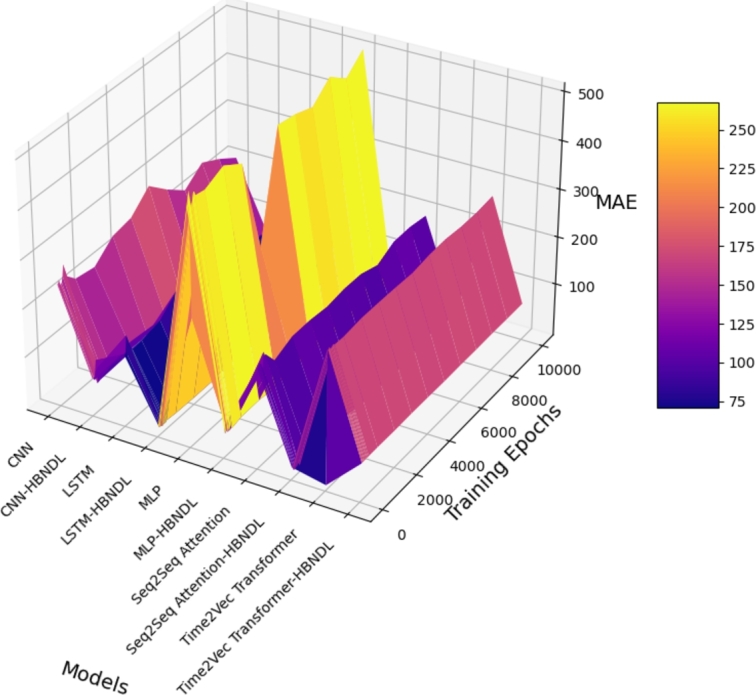
3D surface plot of comparing the impact of the training epochs on MAE for the models.

Fig. 14 presents a comparison of the RMSE for each model across a range of training epochs from 50 to 10,000, while Fig. 15 provides a comparison of the MAE among the models. Our experimental findings indicate that the prediction accuracy of models trained within the HBNDL framework, specifically the LSTM-HBNDL and Seq2Seq Attention-HBNDL models, surpasses that of other models. Notably, the LSTM-HBNDL model achieves an RMSE of 7.64 and an MAE of 5.67. Additionally, the Seq2Seq Attention-HBNDL model yields an RMSE of 11.30 and an MAE of 7.89. The enhancement introduced by HBNDL becomes more pronounced with the increase in training epochs, signifying the framework's particular effectiveness for models requiring prolonged learning processes. Collectively, these experimental results underscore the significant positive influence of the HBNDL framework on the forecast accuracy of various models, affirming its adaptability and efficacy across different training epochs. This highlights HBNDL's value as a beneficial addition to enhancing the performance of forecasting models in diverse scenarios.

This study offers several key advantages in addressing the challenges of predicting EV charging demand. The proposed HBNDL framework combines queuing theory, Bayesian networks, and deep learning techniques to model charging pile reliability and forecast EV charging demand across various conditions. By incorporating meteorological factors and charging pile failures, the model provides a comprehensive approach that enhances prediction accuracy and adaptability over short-, medium-, and long-term periods. Additionally, the use of a decade's worth of real-world transaction data from Palo Alto strengthens the model's robustness and practical relevance. This data-driven foundation ensures that the findings are grounded in real operational patterns, making the model highly applicable for optimizing and planning EV charging infrastructure. Further improvements could expand the model's applicability. Although the framework successfully integrates key variables such as weather and pile failure rates, incorporating additional factors like user behavior variations, economic incentives, and policy shifts could further enhance its adaptability to different regions and evolving EV charging environments. In addition, optimizing the computational demands of the deep learning components could improve the model's feasibility for real-time applications.

Deploying the proposed hybrid Bayesian network and deep learning model in real-world systems poses several challenges, including data collection, privacy and security concerns, and real-time data processing. Our model is designed to process large-scale time-series data and make predictions based on real-time inputs. For successful implementation, the system must efficiently manage real-time data acquisition and execute the model within a charging network environment. One of the key challenges is ensuring the availability of high-quality, real-time data from EV charging stations. The model requires extensive historical data, including charging demand, transaction records, and environmental factors such as temperature and rainfall. However, access to specific data types, including user charging behavior and location-specific data, may be restricted due to privacy regulations and regional legal constraints. While these challenges present hurdles to the practical application of the model, they also underscore the complexity of real-world EV charging systems. Addressing these issues through ongoing research, model refinement, and infrastructure improvements is crucial to unlocking the model's full potential. Despite these difficulties, the integration of probabilistic reasoning and time-series forecasting in this model provides substantial benefits for optimizing EV infrastructure and forecasting demand.

## 5 Conclusions and future research

This study presents a robust analytical framework that integrates queuing network and Bayesian network models to enhance the accuracy and reliability of EV charging demand predictions. By incorporating meteorological factors and charging pile failure rates, our hybrid Bayesian network-based deep learning system architecture significantly improves infrastructure planning and optimization. The key contributions of this research include developing a comprehensive system model to estimate EV charging pile reliability, designing two algorithms to evaluate charging station usage and reliability, and utilizing extensive transaction data and climate analysis to understand the impact of environmental conditions on charging demand. Our extensive experiments validate the adaptability and robustness of the HBNDL framework, demonstrating significant improvements in prediction accuracy across short-, medium-, and long-term scenarios. Integrating queuing theory and Bayesian network models with deep learning techniques underscores the framework's effectiveness in enhancing the reliability of EV charging infrastructure and predicting charging demand more accurately.

This study introduces a novel framework that integrates queuing network models, Bayesian networks, and deep learning techniques to predict EV charging demand. However, several limitations must be acknowledged. First, the study relies primarily on historical data from a single geographic region—Palo Alto, California. This regional limitation may affect the generalizability of the findings to other regions with different environmental conditions, population densities, and EV adoption rates. Future research could address this issue by applying the proposed framework to other geographic areas with varying climatic and infrastructural characteristics. Second, while the hybrid Bayesian Network-based deep learning (HBNDL) framework incorporates meteorological factors and charging pile reliability, it does not explicitly account for the influence of evolving policies, economic incentives, or changes in consumer behavior on EV charging demand. These factors, which can significantly impact charging infrastructure utilization, present opportunities for further research and model refinement. Despite these limitations, this study offers a valuable foundation for future research aimed at enhancing the accuracy and reliability of EV charging demand forecasts. Expanding the analysis to include additional variables would allow the HBNDL framework to evolve and better address the growing complexities of EV infrastructure planning.

Future research can build on this foundation by exploring several key areas. First, extending the model to incorporate additional factors such as varying charging behaviors, economic incentives, and policy changes can provide a more comprehensive understanding of EV charging dynamics. Second, applying this framework to different geographical locations and charging networks can validate its generalizability and adaptability. Third, integrating real-time data streams and advanced machine learning techniques, such as reinforcement learning, can further improve the model's responsiveness and predictive accuracy in dynamic environments. Finally, investigating the socio-economic impacts of optimized EV charging infrastructure on urban mobility and sustainability can provide valuable insights for policymakers and stakeholders in the transition to electric mobility.

## Ethical approval statement

This article does not involve any ethical concerns.

## Human experiments

Not applicable.

## Animal experiments

Not applicable.

## Funding

This work was supported by the 10.13039/100020595National Science and Technology Council of Taiwan under grant numbers NSTC 112-2221-E-130-009- and NSTC 113-2221-E-130-003-.

## Declaration of Competing Interest

The authors declare the following financial interests/personal relationships which may be considered as potential competing interests: David Chunhu Li reports financial support was provided by 10.13039/100020595National Science and Technology Council of Taiwan. David Chunhu Li has patent issued to Ming Chuan University. If there are other authors, they declare that they have no known competing financial interests or personal relationships that could have appeared to influence the work reported in this paper.

## Data Availability

Data will be made available on request. For requesting data, please write to the corresponding author.
